# Investigating the role of the exposome on neuropathology and biochemical phenotypes in Alzheimer’s disease

**DOI:** 10.1002/alz70861_108874

**Published:** 2025-12-23

**Authors:** Gabriela M. Garofalo, Joseph S. Reddy, Michael G. Heckman, Stephanie R Oatman, Xue Wang, Thuy T. Nguyen, Melissa E. Murray, Takahisa Kanekiyo, Dennis W. Dickson, Minerva M. Carrasquillo, Mariet Allen, Nilüfer Ertekin‐Taner

**Affiliations:** ^1^ Mayo Clinic, Jacksonville, FL USA; ^2^ Department of Neuroscience, Mayo Clinic Florida, Jacksonville, FL USA; ^3^ Department of Laboratory and Medicine, Mayo Clinic, Jacksonville, FL USA; ^4^ Department of Neurology, Mayo Clinic, Jacksonville, FL USA

## Abstract

**Background:**

Individual environmental exposures, the exposome, can play an influential role in the development and progression of neurodegenerative diseases such as AD. The protein components of the main AD neuropathologies, amyloid (Aβ) plaques and tau neurofibrillary tangles (NFTs), can be measured using immunohistochemical and biochemical approaches. The main genetic risk factor for AD is the *APOE*e4 allele. APOE levels can also be measured in the brain. We hypothesize that social determinants of health, specifically alcohol and tobacco consumption, and the area deprivation index (ADI), could play a considerable role in severity of neuropathology and brain biochemical phenotypes of AD.

**Method:**

We evaluated 478 neuropathologically characterized AD donors, from the Mayo Clinic Brain Bank. 469 samples had available biochemical measures (APOE, Aβ40, Aβ42, tau, and phospho‐tau (Thr231)), collected across three isolated fractions (soluble (TBS), membrane‐bound (TX), and insoluble (FA)) of the superior temporal gyrus. Exposome variables were extracted from the available records. ADI from the Neighborhood Atlas® version from the year 2015 was obtained from de‐identified addresses. ADI, smoking and alcohol were available for 263 of these donors and were examined for association with biochemical measures using linear regression adjusting for age at death and sex.

**Result:**

Higher ADI levels representing neighborhoods with increased deprivation, were nominally associated (β=0.006, *p*=0.01) with soluble levels of APOE. Alcohol consumption was associated with lower levels of membrane bound APOE (β=‐0.23, *p*=0.04). Analysis with neuropathological variables including CAA (*N* =454), Braak (*N* =454), Thal (*N* =454), Hippocampal sclerosis (*N* =453), Lewy bodies (*N* =455) will also be performed.

**Conclusion:**

Different components of the social exposome may impact levels of core AD proteins. Additional analyses in expanded cohorts to further explore the associations with neuropathology are ongoing. We expect that understanding how the social exposome impacts specific aspects of neurodegenerative diseases will provide key insights into underlying, potentially modifiable, mechanisms of disease.

